# (*Z*)-2-Meth­oxy-*N*-[(5-nitro­thio­phen-2-yl)methyl­idene]aniline

**DOI:** 10.1107/S160053681200044X

**Published:** 2012-01-14

**Authors:** Nihal Kan Kaynar, Sümeyye Gümüs, Erbil Ağar, Orhan Büyükgüngör, Metin Yavuz

**Affiliations:** a Department of Physics, Faculty of Arts & Science, Ondokuz Mayis University, TR-55139 Kurupelit-Samsun, Turkey; b Department of Chemistry, Faculty of Arts & Science, Ondokuz Mayis University, 55139 Samsun, Turkey; c Department of Physics, Faculty of Arts & Science, Ondokuz Mayıs University, TR-55139 Kurupelit-Samsun, Turkey, and, Faculty of Technology, Amasya University, TR-05100 Amasya, Turkey

## Abstract

The dihedral angle between the benzene and thio­phene rings in the title compound, C_12_H_10_N_2_O_3_S, is 27.94 (13)°. An inter­molecular C—H⋯π inter­action contributes to the stability of the crystal structure.

## Related literature

For the biological properties of Schiff bases, see: Barton & Ollis (1979 ▶); Layer (1963 ▶); Ingold (1969 ▶), for their industrial properties, see: Taggi *et al.* (2002 ▶) and for their reaction properties, see: Aydoğan *et al.* (2001 ▶). For related structures, see: Ağar *et al.* (2010 ▶); Tanak *et al.* (2009 ▶); Ceylan *et al.* (2011 ▶).
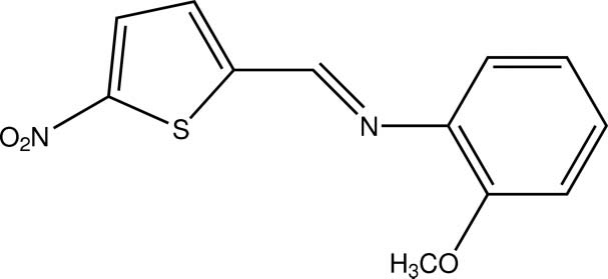


## Experimental

### 

#### Crystal data

C_12_H_10_N_2_O_3_S
*M* *_r_* = 262.28
Orthorhombic, 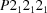

*a* = 6.6825 (6) Å
*b* = 7.7926 (5) Å
*c* = 23.7180 (12) Å
*V* = 1235.09 (15) Å^3^
*Z* = 4
Mo *K*α radiation
μ = 0.26 mm^−1^
*T* = 296 K
0.59 × 0.39 × 0.05 mm

#### Data collection

Stoe IPDS II diffractometer
Absorption correction: integration (*X-RED32*; Stoe & Cie, 2002 ▶) *T*_min_ = 0.974, *T*_max_ = 0.974
5645 measured reflections
2599 independent reflections
1799 reflections with *I* > 2σ(*I*)
*R*_int_ = 0.038

#### Refinement

*R*[*F* ^2^ > 2σ(*F* ^2^)] = 0.037
*wR*(*F* ^2^) = 0.069
*S* = 0.93
2599 reflections
163 parameters
H-atom parameters constrained
Δρ_max_ = 0.12 e Å^−3^
Δρ_min_ = −0.21 e Å^−3^
Absolute structure: Flack (1983 ▶), 1067 Friedel pairs
Flack parameter: −0.04 (8)

### 

Data collection: *X-AREA* (Stoe & Cie, 2002 ▶); cell refinement: *X-AREA*; data reduction: *X-RED32* (Stoe & Cie, 2002 ▶); program(s) used to solve structure: *SHELXS97* (Sheldrick, 2008 ▶); program(s) used to refine structure: *SHELXL97* (Sheldrick, 2008 ▶); molecular graphics: *ORTEP-3 for Windows* (Farrugia, 1997 ▶); software used to prepare material for publication: *WinGX* (Farrugia, 1999 ▶).

## Supplementary Material

Crystal structure: contains datablock(s) I, global. DOI: 10.1107/S160053681200044X/bt5776sup1.cif

Structure factors: contains datablock(s) I. DOI: 10.1107/S160053681200044X/bt5776Isup2.hkl

Supplementary material file. DOI: 10.1107/S160053681200044X/bt5776Isup3.cml

Additional supplementary materials:  crystallographic information; 3D view; checkCIF report

## Figures and Tables

**Table 1:** Hydrogen-bond geometry (Å, °) *Cg*2 is the centroid of the C6–C11 ring.

*D*—H⋯*A*	*D*—H	H⋯*A*	*D*⋯*A*	*D*—H⋯*A*
C10—H10⋯*Cg*2^i^	0.93	2.77	3.605 (3)	149

Symmetry code: (i) 
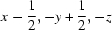
.

**Fig. 1.**
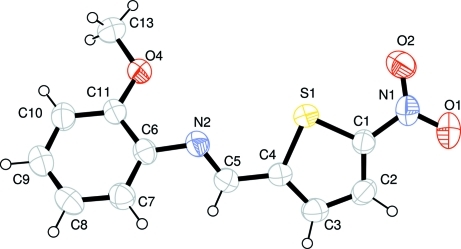
The molecular structure of the title compound, showing the atom-numbering scheme and 50% probability diplacement ellipsoids.

## supplementary crystallographic information

### Comment

Schiff bases, *i.e.*, compounds having a double C=N bond, are used as starting materials in the synthesis of important drugs, such as antibiotics and antiallergic, antiphlogistic, and antitumor substances (Barton *et al.*, 1979; Layer, 1963; Ingold 1969). On the industrial scale, they have a wide range of applications, such as dyes and pigments (Taggi *et al.*, 2002). Schiff bases have also been employed as ligands for the complexation of metal ions (Aydoğan *et al.*, 2001).

The molecular structure of the title compound is shown on Fig. 1. The dihedral angle between the C10—C13/S1 nitrothiophene and the C1—C6 phenyl ring is 27.94 (13)°. The deviation from planarity may be due to steric repulsion between the methylene group and phenyl ring. The length of the C5=N2 double bond is 1.266 (3) Å, slightly shorter than standard 1.28 Å value of a C=N double bond and consistent with related structures (Ağar *et al.*, 2010; Tanak *et al.*, 2009; Ceylan *et al.*, 2011).

The crystal structure is stabilized by an intermolecular C—H···π interaction (C10—H10···*Cg*2). No significant π—π interactions are observed in the crystal structure.

### Experimental

The compound (*Z*)—*N*-(2-methoxyphenyl)-1-(5-nitrothiophen-2-yl)methanimine was prepared by reflux a mixture of a solution containing 5-nitro-2-thiophene-carboxaldehyde (0.0078 g 0.050 mmol) in 20 ml e thanol and a solution containing *o*-Anisidine (0.0062 g 0.050 mmol) in 20 ml e thanol. The reaction mixture was stirred for 1 hunder reflux. The crystals of (*Z*)—*N*-(2-methoxyphenyl)-1-(5-nitrothiophen-2-yl)methanimine suitable for X-ray analysis were obtained from ethylalcohol by slow evaporation (yield % 76; 85–87 °C).

### Refinement

H atoms were positioned geometrically and refined using a riding model, with C—H = 0.93–0.96 Å and *U*_iso_(H) = 1.2–1.5*U*_eq_(C).

### Crystal data

**Table:**

C_12_H_10_N_2_O_3_S	*F*(000) = 544
*M**_r_* = 262.28	*D*_x_ = 1.411 Mg m^−^^3^
Orthorhombic, *P*2_1_2_1_2_1_	Mo *K*α radiation, λ = 0.71073 Å
Hall symbol: P 2ac 2ab	Cell parameters from 6785 reflections
*a* = 6.6825 (6) Å	θ = 2.6–27.2°
*b* = 7.7926 (5) Å	µ = 0.26 mm^−^^1^
*c* = 23.7180 (12) Å	*T* = 296 K
*V* = 1235.09 (15) Å^3^	Plate, yellow
*Z* = 4	0.59 × 0.39 × 0.05 mm

### Data collection

**Table:**

Stoe IPDS II diffractometer	2599 independent reflections
Radiation source: fine-focus sealed tube	1799 reflections with *I* > 2σ(*I*)
graphite	*R*_int_ = 0.038
Detector resolution: 6.67 pixels mm^-1^	θ_max_ = 26.8°, θ_min_ = 2.8°
rotation method scans	*h* = −6→8
Absorption correction: integration (*X-RED32*; Stoe & Cie, 2002)	*k* = −9→9
*T*_min_ = 0.974, *T*_max_ = 0.974	*l* = −29→29
5645 measured reflections	

### Refinement

**Table:**

Refinement on *F*^2^	Secondary atom site location: difference Fourier map
Least-squares matrix: full	Hydrogen site location: inferred from neighbouring sites
*R*[*F*^2^ > 2σ(*F*^2^)] = 0.037	H-atom parameters constrained
*wR*(*F*^2^) = 0.069	*w* = 1/[σ^2^(*F*_o_^2^) + (0.0284*P*)^2^] where *P* = (*F*_o_^2^ + 2*F*_c_^2^)/3
*S* = 0.93	(Δ/σ)_max_ < 0.001
2599 reflections	Δρ_max_ = 0.12 e Å^−^^3^
163 parameters	Δρ_min_ = −0.21 e Å^−^^3^
0 restraints	Absolute structure: Flack (1983), 1067 Friedel pairs
Primary atom site location: structure-invariant direct methods	Flack parameter: −0.04 (8)

### Special details

**Table:**

Experimental. 108 frames, detector distance = 120 mm
Geometry. All e.s.d.'s (except the e.s.d. in the dihedral angle between two l.s. planes) are estimated using the full covariance matrix. The cell e.s.d.'s are taken into account individually in the estimation of e.s.d.'s in distances, angles and torsion angles; correlations between e.s.d.'s in cell parameters are only used when they are defined by crystal symmetry. An approximate (isotropic) treatment of cell e.s.d.'s is used for estimating e.s.d.'s involving l.s. planes.
Refinement. Refinement of *F*^2^ against ALL reflections. The weighted *R*-factor *wR* and goodness of fit *S* are based on *F*^2^, conventional *R*-factors *R* are based on *F*, with *F* set to zero for negative *F*^2^. The threshold expression of *F*^2^ > σ(*F*^2^) is used only for calculating *R*-factors(gt) *etc*. and is not relevant to the choice of reflections for refinement. *R*-factors based on *F*^2^ are statistically about twice as large as those based on *F*, and *R*- factors based on ALL data will be even larger.

### Fractional atomic coordinates and isotropic or equivalent isotropic displacement parameters (Å^2^)

**Table:**

	*x*	*y*	*z*	*U*_iso_*/*U*_eq_	
S1	0.93052 (10)	0.80791 (7)	0.16132 (3)	0.06181 (17)	
O4	0.4298 (3)	0.59180 (17)	0.04012 (7)	0.0711 (5)	
C7	0.5822 (4)	0.2075 (3)	0.11420 (10)	0.0649 (6)	
H7	0.6780	0.1749	0.1405	0.078*	
C11	0.4265 (4)	0.4242 (3)	0.05670 (10)	0.0572 (5)	
C6	0.5750 (4)	0.3765 (3)	0.09543 (9)	0.0568 (6)	
N2	0.7021 (4)	0.5066 (2)	0.11656 (8)	0.0613 (5)	
C1	1.1502 (4)	0.8578 (3)	0.19332 (10)	0.0561 (6)	
O1	1.3451 (3)	1.0633 (2)	0.23403 (9)	0.0968 (7)	
C8	0.4476 (5)	0.0878 (3)	0.09384 (11)	0.0735 (7)	
H8	0.4546	−0.0255	0.1060	0.088*	
N1	1.1877 (4)	1.0325 (3)	0.20966 (9)	0.0695 (6)	
C5	0.8791 (4)	0.4701 (3)	0.13135 (10)	0.0614 (6)	
H5	0.9268	0.3592	0.1259	0.074*	
C9	0.3052 (5)	0.1357 (3)	0.05614 (12)	0.0744 (8)	
H9	0.2147	0.0546	0.0428	0.089*	
C4	1.0087 (3)	0.5975 (3)	0.15657 (11)	0.0562 (6)	
O2	1.0599 (4)	1.1402 (2)	0.19817 (8)	0.0883 (6)	
C10	0.2928 (4)	0.3033 (3)	0.03729 (10)	0.0671 (6)	
H10	0.1944	0.3343	0.0115	0.081*	
C3	1.1934 (4)	0.5742 (3)	0.17897 (11)	0.0675 (7)	
H3	1.2582	0.4686	0.1798	0.081*	
C2	1.2765 (4)	0.7248 (3)	0.20070 (11)	0.0685 (7)	
H2	1.4013	0.7323	0.2178	0.082*	
C13	0.2741 (5)	0.6489 (3)	0.00482 (13)	0.0897 (9)	
H13A	0.2933	0.7682	−0.0037	0.135*	
H13B	0.1481	0.6339	0.0236	0.135*	
H13C	0.2748	0.5837	−0.0295	0.135*	

### Atomic displacement parameters (Å^2^)

**Table:**

	*U*^11^	*U*^22^	*U*^33^	*U*^12^	*U*^13^	*U*^23^
S1	0.0602 (3)	0.0558 (3)	0.0694 (4)	0.0056 (3)	−0.0122 (4)	0.0013 (3)
O4	0.0705 (11)	0.0537 (8)	0.0889 (13)	−0.0040 (9)	−0.0184 (12)	0.0056 (8)
C7	0.0688 (15)	0.0610 (12)	0.0650 (14)	−0.0041 (14)	0.0017 (15)	0.0057 (12)
C11	0.0587 (13)	0.0531 (11)	0.0598 (14)	−0.0041 (12)	0.0017 (14)	−0.0040 (9)
C6	0.0613 (14)	0.0544 (11)	0.0548 (14)	−0.0042 (12)	0.0049 (14)	−0.0065 (9)
N2	0.0669 (15)	0.0554 (10)	0.0615 (12)	−0.0027 (10)	−0.0049 (11)	−0.0060 (9)
C1	0.0587 (15)	0.0552 (12)	0.0543 (14)	−0.0048 (10)	−0.0054 (12)	0.0014 (10)
O1	0.0939 (17)	0.0998 (13)	0.0967 (15)	−0.0252 (12)	−0.0188 (13)	−0.0225 (11)
C8	0.087 (2)	0.0523 (12)	0.0808 (19)	−0.0052 (15)	0.0142 (18)	0.0050 (12)
N1	0.0762 (16)	0.0724 (14)	0.0598 (14)	−0.0166 (13)	−0.0058 (12)	−0.0045 (10)
C5	0.0612 (19)	0.0548 (12)	0.0681 (16)	0.0022 (11)	−0.0007 (13)	−0.0027 (11)
C9	0.0749 (18)	0.0668 (15)	0.0813 (19)	−0.0151 (14)	0.0057 (18)	−0.0075 (13)
C4	0.0531 (14)	0.0539 (11)	0.0616 (15)	0.0036 (9)	−0.0017 (12)	−0.0006 (11)
O2	0.1080 (15)	0.0631 (10)	0.0937 (14)	0.0073 (12)	−0.0122 (15)	−0.0038 (9)
C10	0.0635 (15)	0.0672 (14)	0.0706 (16)	−0.0065 (13)	−0.0015 (14)	−0.0088 (13)
C3	0.0599 (17)	0.0611 (13)	0.0816 (19)	0.0080 (13)	−0.0018 (14)	0.0015 (12)
C2	0.0580 (15)	0.0760 (16)	0.0714 (16)	−0.0016 (13)	−0.0133 (13)	0.0070 (13)
C13	0.081 (2)	0.0754 (16)	0.112 (2)	0.0061 (15)	−0.0289 (19)	0.0128 (15)

### Geometric parameters (Å, °)

**Table:**

S1—C1	1.698 (2)	C8—H8	0.9300
S1—C4	1.725 (2)	N1—O2	1.228 (3)
O4—C11	1.364 (2)	C5—C4	1.447 (3)
O4—C13	1.408 (3)	C5—H5	0.9300
C7—C8	1.383 (4)	C9—C10	1.383 (3)
C7—C6	1.391 (3)	C9—H9	0.9300
C7—H7	0.9300	C4—C3	1.356 (3)
C11—C10	1.378 (3)	C10—H10	0.9300
C11—C6	1.402 (3)	C3—C2	1.397 (3)
C6—N2	1.414 (3)	C3—H3	0.9300
N2—C5	1.266 (3)	C2—H2	0.9300
C1—C2	1.348 (3)	C13—H13A	0.9600
C1—N1	1.437 (3)	C13—H13B	0.9600
O1—N1	1.224 (3)	C13—H13C	0.9600
C8—C9	1.358 (4)		
			
C1—S1—C4	89.14 (11)	C4—C5—H5	119.3
C11—O4—C13	117.5 (2)	C8—C9—C10	120.9 (2)
C8—C7—C6	120.3 (2)	C8—C9—H9	119.5
C8—C7—H7	119.9	C10—C9—H9	119.5
C6—C7—H7	119.9	C3—C4—C5	127.9 (2)
O4—C11—C10	124.7 (2)	C3—C4—S1	112.20 (18)
O4—C11—C6	115.5 (2)	C5—C4—S1	119.86 (17)
C10—C11—C6	119.8 (2)	C11—C10—C9	119.9 (2)
C7—C6—C11	119.0 (2)	C11—C10—H10	120.0
C7—C6—N2	123.0 (2)	C9—C10—H10	120.0
C11—C6—N2	117.87 (19)	C4—C3—C2	113.2 (2)
C5—N2—C6	119.9 (2)	C4—C3—H3	123.4
C2—C1—N1	125.7 (2)	C2—C3—H3	123.4
C2—C1—S1	115.04 (17)	C1—C2—C3	110.4 (2)
N1—C1—S1	119.23 (18)	C1—C2—H2	124.8
C9—C8—C7	120.0 (2)	C3—C2—H2	124.8
C9—C8—H8	120.0	O4—C13—H13A	109.5
C7—C8—H8	120.0	O4—C13—H13B	109.5
O1—N1—O2	124.6 (2)	H13A—C13—H13B	109.5
O1—N1—C1	117.6 (2)	O4—C13—H13C	109.5
O2—N1—C1	117.8 (2)	H13A—C13—H13C	109.5
N2—C5—C4	121.3 (2)	H13B—C13—H13C	109.5
N2—C5—H5	119.3		
			
C13—O4—C11—C10	6.3 (4)	S1—C1—N1—O2	−2.0 (3)
C13—O4—C11—C6	−175.0 (2)	C6—N2—C5—C4	−175.9 (2)
C8—C7—C6—C11	1.5 (4)	C7—C8—C9—C10	0.4 (4)
C8—C7—C6—N2	176.9 (2)	N2—C5—C4—C3	173.5 (3)
O4—C11—C6—C7	−179.7 (2)	N2—C5—C4—S1	−5.6 (3)
C10—C11—C6—C7	−1.0 (3)	C1—S1—C4—C3	0.1 (2)
O4—C11—C6—N2	4.6 (3)	C1—S1—C4—C5	179.4 (2)
C10—C11—C6—N2	−176.7 (2)	O4—C11—C10—C9	178.8 (2)
C7—C6—N2—C5	33.7 (4)	C6—C11—C10—C9	0.2 (4)
C11—C6—N2—C5	−150.8 (2)	C8—C9—C10—C11	0.1 (4)
C4—S1—C1—C2	−0.4 (2)	C5—C4—C3—C2	−179.0 (2)
C4—S1—C1—N1	179.1 (2)	S1—C4—C3—C2	0.2 (3)
C6—C7—C8—C9	−1.2 (4)	N1—C1—C2—C3	−178.9 (2)
C2—C1—N1—O1	−2.5 (4)	S1—C1—C2—C3	0.6 (3)
S1—C1—N1—O1	178.03 (18)	C4—C3—C2—C1	−0.5 (3)
C2—C1—N1—O2	177.5 (2)		

### Hydrogen-bond geometry (Å, °)

**Table:**

*Cg*2 is the centroid of the C6–C11 ring.

**Table:**

*D*—H···*A*	*D*—H	H···*A*	*D*···*A*	*D*—H···*A*
C10—H10···*Cg*2^i^	0.93	2.77	3.605 (3)	149

Symmetry codes: (i) *x*−1/2, −*y*+1/2, −*z*.
